# Neonatal Pain, Opioid, and Anesthetic Exposure; What Remains in the Human Brain After the Wheels of Time?

**DOI:** 10.3389/fped.2022.825725

**Published:** 2022-05-11

**Authors:** Gerbrich E. van den Bosch, Dick Tibboel, Jurgen C. de Graaff, Hanan El Marroun, Aad van der Lugt, Tonya White, Monique van Dijk

**Affiliations:** ^1^Intensive Care and Department of Pediatric Surgery, Erasmus MC-Sophia Children's Hospital, Rotterdam, Netherlands; ^2^Division of Neonatology, Department of Pediatrics, Erasmus Medical Center (MC)-Sophia Children's Hospital, Rotterdam, Netherlands; ^3^Department of Anesthesiology, Erasmus MC-Sophia Children's Hospital, Rotterdam, Netherlands; ^4^Department of Child and Adolescent Psychiatry/Psychology, Erasmus MC-Sophia Children's Hospital, Rotterdam, Netherlands; ^5^Department of Psychology, Education and Child Studies, Erasmus University, Rotterdam, Netherlands; ^6^Department of Radiology, Erasmus MC, Rotterdam, Netherlands

**Keywords:** brain, children, human, neuroimaging, opioids, pain, anesthesia

## Abstract

**Objective:**

To evaluate possible negative long-term effects of neonatal exposure to pain, opioids and anesthetics in children and adolescents.

**Study Design:**

We studied five unique groups of children recruited from well-documented neonatal cohorts with a history of neonatal exposure to pain, opioids or anesthetics at different points along the continuum from no pain to intense pain and from no opioid exposure to very high opioid exposure in the presence or absence of anesthetics. We evaluated children who underwent major surgery (group 1 and 2), extracorporeal membrane oxygenation (group 3), preterm birth (group 4) and prenatal opioid exposure (group 5) in comparison to healthy controls. Neuropsychological functioning, thermal detection and pain thresholds and high-resolution structural and task-based functional magnetic resonance imaging during pain were assessed. In total 94 cases were included and compared to their own control groups.

**Results:**

Children and adolescents in groups 3 and 5 showed worse neuropsychological functioning after high opioid exposure. A thicker cortex was found in group 1 (pain, opioid and anesthetic exposure) in only the left rostral-middle-frontal-cortex compared to controls. We found no differences in other brain volumes, pain thresholds or brain activity during pain in pain related brain regions between the other groups and their controls.

**Conclusions:**

No major effects of neonatal pain, opioid or anesthetic exposure were observed in humans 8–19 years after exposure in early life, apart from neuropsychological effects in the groups with the highest opioid exposure that warrants further investigation. Studies with larger sample sizes are needed to confirm our findings and test for less pronounced differences between exposed and unexposed children.

## Introduction

Not only early exposure to anesthetics, but also pain and opioids are associated with negative outcome at least in animals (1, 2). These consist of cell death in the brain and alterations in pain sensitivity after neonatal pain and degeneration of red neurons, apoptosis in several brain regions, impaired cued fear extinction, and impaired cognitive functioning after neonatal opioid exposure (3–10). While these negative effects occurred in the absence of pain, protective effects of opioid exposure in the presence of pain are observed as well (3, 11, 12).

**Figure 1:**
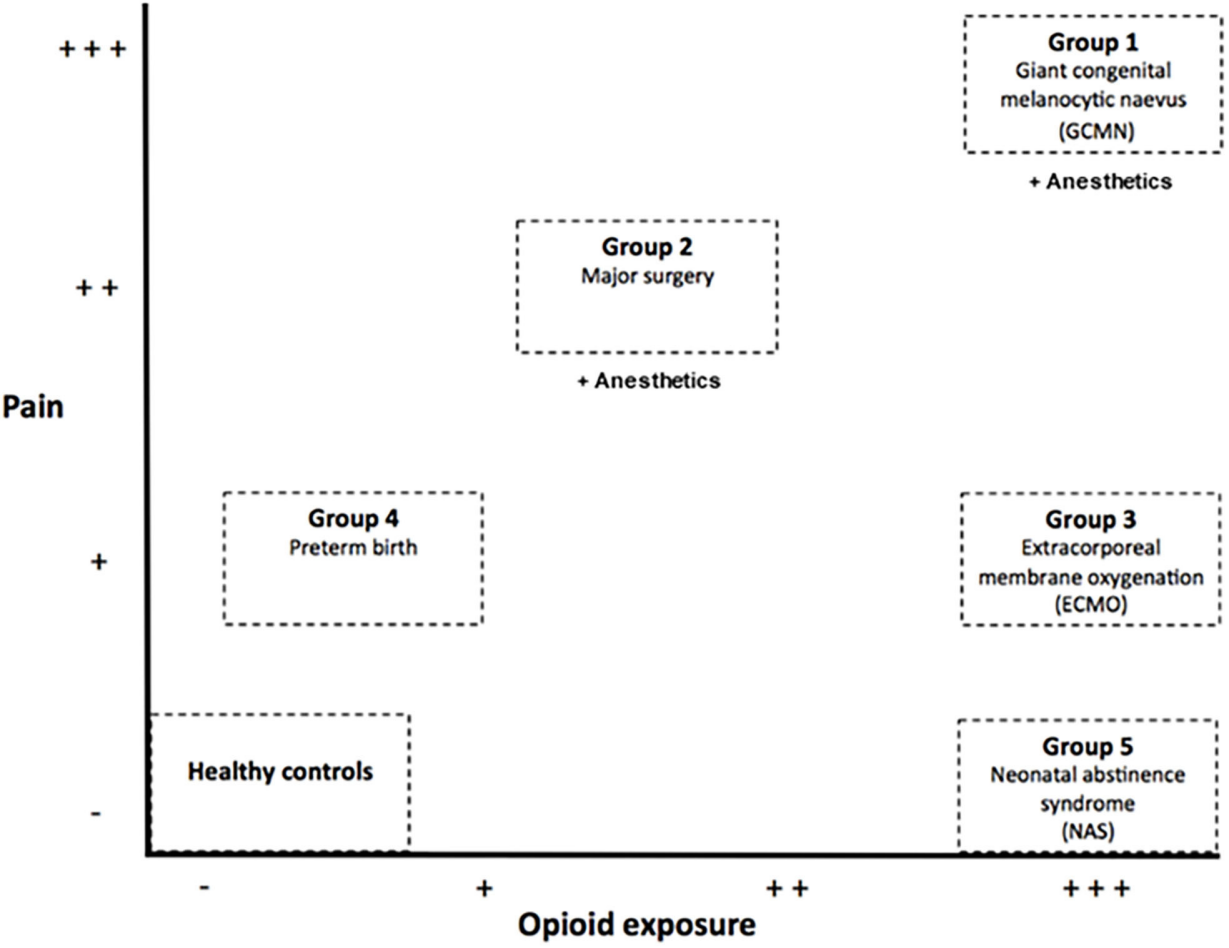
Study groups.

In humans with major congenital anomalies there is a clinical need for surgery in the neonatal period, resulting in the combination of potential pain, opioid and anesthetic exposure. However, studies on the potential long-term effects of pain, anesthetics and opioids with respect to neurodevelopment in humans are scarce and show contradictory results. Possibly because studies in children are not systematically in design and mainly investigate only very specific groups of patients such as extremely preterm born children and former newborns after thoracotomy (13, 14). To obtain a comprehensive view on the potential individual and combined effect of these factors in human, we studied five unique well-documented groups, which reflect exposure to pain, opioids and anesthetics at different points along the continuum from no pain to intense pain and from no opioid exposure to very high opioid exposure in the presence or absence of anesthetics (Figure 1). We hypothesize that children with a history of neonatal pain, opioid and/or anesthetic exposure will experience overall far reaching negative long-term consequences on several domains such as pain sensitivity, brain morphology, brain functioning and neuropsychological performance. We expect the most negative effects in children who received high dosages of opioids in the absence of severe pain, as suggested by animal studies.

## Materials and Methods

### Patients and Methods

#### Ethics Approval

The study was performed at Erasmus MC in Rotterdam and was approved by the Institutional Review Board at the Erasmus MC (MEC-2010-299). Written informed consent was obtained from the parents and assent was obtained from the participants themselves. Recruitment took place from March 2011 to December 2013.

#### Patient Population

##### Cases

Participants were recruited from 5 different cohorts with a history of exposure to neonatal pain, opioids or anesthetics. All selected children were at least 8 years old at time of inclusion. We did not conduct a formal power analysis since brain activation during pain was our main outcome measure and multivariate effect estimates are in general difficult to estimate in fMRI studies. The sample size necessary to obtain adequate power for our fMRI experiment was extracted from two prior studies, which demonstrated differences for pain and brush and cold stimuli with samples containing 8 to 9 children (15, 16). However, due to collecting more groups, with the potential for greater heterogeneity, our goal was to recruit at least 15 participants per group, with each being matched with a control group.

##### Group 1—Giant Congenital Melanocytic Naevus

Children who required a very painful exchochleation procedure of the skin of up till 30% of their body surface area (BSA) in the first weeks of life due to a GCMN, with extreme pain and high opioid exposure (range perioperative 241-14973 mcg/kg) in combination with exposure to anesthetics during surgery and ICU admission after surgery in our hospital. Twenty-four children between 8 and 18 years of age at the moment of inclusion were eligible and invited for our study. The families of five children declined participation. Two other children had permanent braces and could not participate in the MRI study. The families of these two children chose not to participate solely in the non-MRI tests. Three children were lost to follow up. Thus, 14 GCMN children were included in this study.

##### Group 2—Major Surgery

The major surgery group consists of children who participated in a double blind RCT as neonate. The original study was conducted between 1995 and 1998 in Rotterdam. Eligible for the present study were 62 children who required major surgery in the first month of life [e.g. abdominal, non-cardiac thoracic (17)] with relatively lower pain intensity compared to group 1 and normal opioid exposure (cumulative dose of 10 mcg/kg/h in the first 24 h) in combination with exposure to anesthetics during surgery. Seven cases had been lost to follow-up, and 23 had a known contra-indication for participation in a neuroimaging and neuropsychological study. Thirty-two children were eligible and invited. Eight families could not be reached by phone and another 14 families declined participation, mostly because the adolescent felt not inclined. The remaining 10 cases were willing to participate and were included in our study.

##### Group 3—Neonatal Extracorporeal Membrane Oxygenation

For the ECMO group we invited children who as neonates had received venoarterial ECMO treatment in our hospital (18), and received high dosages of opioids for extended periods to avoid accidental ECMO decannulation, generally in the absence of major pain as ECMO cannulation should be considered as minor surgery. Of the 165 children, 44 (27%) had died. Excluded were 15 children who did not join our follow-up program, and 46 children with contra-indications for participation in a MRI study or neuropsychological assessment. The remaining 60 children were invited. Six families were not traceable and 17 declined participation. One child turned out to have permanent braces and was given the opportunity to participate in the non-MRI tests, but the family declined. The remaining 36 children were included in our study.

##### Group 4—Preterm Born Children

The preterm born children were recruited from a cohort of preterm born children who at neonatal age had participated in an RCT comparing continuous infusion of morphine with placebo with repeated short periods of exposure to pain and hardly opioids (19, 20). For this specific cohort no twins or triplets were included. Twenty-two families were invited. One child was lost to follow-up and two families declined. The other 19 were included.

##### Group 5—Neonatal Abstinence Syndrome (NAS)

For the NAS cohort we selected children form our electronic patient system who were admitted to the Erasmus MC-Sophia Children's Hospital in Rotterdam from October 1993 to May 2005 for treatment of NAS due to prenatal exposure to opioid related substances (morphine, methadone, heroin) taken as drugs of abuse by their mothers. Eighty children were found, but one died, 17 were lost to follow-up and 19 were excluded from this study for several reasons including medical problems, such as severe hearing loss, since these children could not properly participate in the different tests. A letter with relevant information was sent to the remaining 43 cases. Of these children, 17 could not be reached by phone, and 10 cases declined participation. One case was excluded because of previously unknown intellectual disabilities. The remaining 15 cases were included (see also Supplementary Figure 1).

##### Controls

Healthy controls were recruited in two ways. First, we asked all participating families whether they could recommend someone in the age range of 8–18 years. Second, we mailed invitation letters to parents of children attending primary schools in Rotterdam. Each case group was compared to its own controls based on sampling from the total control group. Controls were selected within a comparable age range. Controls were term born children without a history of admission to a (Neonatal) Intensive Care Unit (NICU) and without neonatal opioid exposure or significant pain experience, analgesic or sedative use in the first year of life.

#### Exclusion Criteria

Candidates were screened for exclusion criteria and contra-indications for participation in an MRI study; brain abnormalities found on previous ultrasounds, CT, or MR scans including any grade of intraventricular hemorrhage (IVH), periventricular leukomalacia (PVL) and subependymal cysts since brain abnormalities could possibly influence brain functioning during pain or MRI segmentation resulting in unreliable brain measures), diagnosed mental or neurologic disorders, or gross motor or sensory disabilities (such as blindness or deafness) since these children could not properly understand the procedure and brain abnormalities would influence our structural and functional MRI results. Additional exclusion criterion for children from the preterm born cohort was being a twin or triplet and for controls a history of severe early pain or intensive care admission.

#### Procedure

All children and adolescents were tested using a comparable systematic study design. Cases and controls first underwent a neuropsychological assessment. Subsequently, thermal detection- and pain thresholds were determined. Finally, a structural MRI scan and two task-based functional MRI scans with thermal pain stimuli were obtained.

#### Neuropsychological Assessment

The NEPSY-II-NL neuropsychological test was conducted in children up till 16 years of age (Pearson, Amsterdam), which is a Dutch translation of the North American NEPSY-II (21). Children between 8 and 12 years of age performed nine subtests including domains of attention and executive functioning, language, memory and learning, sensorimotor functioning, and visuospatial processing. Older participants performed only six of these subtests due to the age limit of the three other tests.

#### Examination of Detection and Pain Thresholds

To determine detection- and pain thresholds we used the Thermal Sensory Analyzer-II (TSA-II, Medoc Advanced Medical systems, Israel). See the Supplementary File for detailed information on the examination of detection and pain thresholds.

#### Image Acquisition and Functional MRI Block Paradigm

Detailed information on the image acquisition and functional MRI paradigm are given in the Supplementary File including the Supplementary Figure 2—Block Paradigm.

#### Structural Imaging Analysis

We used the FreeSurfer image analysis suite version 5.1.0 (http://surfer.nmr.mgh.harvard.edu/) for cortical reconstruction and volumetric segmentation. See the Supplementary File for detailed information on the structural imaging analysis.

#### Functional Imaging Analysis

For functional MRI analyses (fMRI), we used a combination of Analysis of Functional Neuroimages (AFNI, http://afni.nimh.nih.gov/) (22) and FSL's FMRIB's Software Library (FSL 5.0, FMRIB Software Library; FMRIB, Functional Magnetic Resonance Imaging of the Brain; http://www.fmrib.ox.ac.uk/fsl/) (23). See the Supplementary File for detailed information on the functional imaging analysis.

#### Data Analysis

MRI analyses are described in detail in the Supplementary File. For the analyses on neuropsychological functioning we used the Mann-Whitney U and ANCOVA tests. For the analyses on detection- and pain thresholds we used Independent samples *T*-test for continuous and Fisher's Exact and χ^2^-tests for categorical variables. We corrected for multiple testing using Bonferroni correction. Correlations between morphine exposure in our largest (ECMO) cohort were compared to detection- and pain thresholds, neuropsychological outcome, and brain volumes were determined using Spearmans' rank order correlation coefficient. A *p*-value of 0.05 or less was considered statistically significant.

Each case group was compared to its own controls. We included 5 different heterogeneous clinical case groups with differences in pain and opioid exposure. Because of the heterogeneity between the groups in, for example gestation age, age at testing and underlying disease, we did not want to make assumptions about the relationship between pain, opioid exposure and the underlying neurobiology in the pooled sample. Pooling of the groups is associated with the possible incorrect assumption that opioids and pain at different times during development and in heterogeneous groups will result in similar long-term outcomes.

## Results

### Study Population

Background characteristics were retrieved from the medical records and are presented in Table 1 and with more detail in the Table 2.

**Table 1:** Background characteristics per group.

**(a). Group 1–GCMN** ***N* = 14**		
General characteristics		
Gestational age in weeks (median, range)*		40.4 (35.3–41.6)
Birth weight (grams, median, range)*		3,540 (2,500–5,000)
Pharmacological data		
Total use of IV morphine perioperative in mcg/kg (median, range)**		2,766 (241–14,973)
Total use of IV midazolam postoperatively in mg/kg (median, range)***		9.7 (0–58)
**(b). Group 2–Major surgery** ***N** **=*** **10**		
General characteristics		
Gestational age in weeks (median, range)		38.3 (33.2–41.0)
Birth weight (grams, median, range)		3,178 (2,200–4,230)
Pharmacological data		
Additional morphine administration first 24 h [*n* (%) yes]		3 (30)
Cumulative morphine dose first 24 h (μg kg^−1^ h^−1^, median, range)		10.0 (10.0–11.2)
**(c). Group 3–ECMO** ***N** **=*** **36**		
General characteristics		
Gestational age in weeks (median, range)		40 (37–43)
Birth weight in grams (median, range)		3,535 (2,300–4,985)
**Pharmacological data**		
Maximum morphine exposure prior to ECMO (*n*, %)*	None	2 (6)
	10 mcg/kg/h or less	16 (47)
	11–20 mcg/kg/h	12 (35)
	More than 20 mcg/kg/h	4 (12)
Maximum morphine exposure during ECMO (*n*, %)*	None	3 (9)
	10 mcg/kg/h or less	14 (41)
	11–20 mcg/kg/h	9 (26)
	More than 20 mcg/kg/h	8 (24)
Maximum morphine exposure after ECMO (*n*, %)**	None	4 (11)
	10 mcg/kg/h or less	15 (43)
	11–20 mcg/kg/h	6 (17)
	More than 20 mcg/kg/h	10 (29)
Maximum sedative exposure prior to ECMO (*n*, %)*	None	16 (47)
	0.1 mg/kg/h or less	11 (32)
	0.11–0.2 mg/kg/h	6 (18)
	More than 0.2 mg/kg/h	1 (3)
Maximum sedative exposure during ECMO (*n*, %)***	None	6 (18)
	0.1 mg/kg/h or less	11 (33)
	0.11–0.2 mg/kg/h	9 (27)
	More than 0.2 mg/kg/h	7 (21)
Maximum sedative exposure after ECMO (*n*, %)***	None	1 (3)
	0.1 mg/kg/h or less	12 (36)
	0.11–0.2 mg/kg/h	10 (30)
	More than 0.2 mg/kg/h	10 (30)
Duration of morphine exposure (%)**	<1 week	6 (17)
	1 week−1 month	25 (71)
	More than 1 month	4 (11)
Duration of sedative exposure (%)**	<1 week	7 (20)
	1 week−1 month	23 (66)
	More than 1 month	5 (14)
Methadone treatment in the first year of life for weaning from opioids (% yes)****		5 (14)
**(d). Group 4–Preterm born children** ***N** **=*** **19**		
General characteristics		
Gestational age in weeks (median, range)		31.1 (26.1–36.3)
Birth weight (grams, median, range)		1,415 (675–2,895)
Pharmacological data		
Morphine administration (% yes)		78.9
Cumulative use of IV morphine in the first 28 days in mcg/kg (median, range)		393.6 (0–4873)
**(e). Group 5–NAS** *N =* 15		
Birth characteristics		
Gestational age, weeks (median, IQR)		38 (36–41)
Birth weight, in grams (median, IQR)		2,935 (2,400–3,215)
**Pharmacological data**		
Prenatal exposure to Methadone (*n*, %)		13 (87)
Prenatal exposure to Heroine (*n*, %)		12 (80)
Prenatal opioid exposure in combination with:	Cocaine (*n*, %) Benzodiazepines (*n*, %)	13 (87) 1 (7)
Phenobarbital treatment (*n*, %)		14 (93)

(a). *Based on n = 8 due to missing data.

**In 4 children the medical record was incomplete and therefore the actual morphine dose could be higher than reported.

***In 2 children the medical record was incomplete and therefore the actual midazolam dose could be higher than reported.

(b). *The surgical stress score measures the severity of surgical stress in neonates and has a range from 3–22, for more information see van Dijk et al. (17).

**Based on n = 9 since one child was removed from the original RCT after 6 h postoperatively due to incidental removal of the arterial line.

(c). *Based on n = 34 due to missing date.

**Based on n = 35 due to missing data.

***Based on n = 33 due to missing data.

****Methadone was started at a median age of 30 days (range 20 to 47 days) at a median daily dose of 4 mg (range 2 to 9 mg).

**Table 2:** Additional background characteristics per group.

**(a). Group 1–GCMN** ***N =* 14**		
Surgery		
Age at time of surgery in days (median, range)		31 (10–53)
Total body surface area in % (median, range)*		18 (5–30)
Location of the Tierfell Naevus (%)	Back	35.7
	Face or skull	28.6
	Chest and arm(s)	14.3
	Chest and leg(s)	14.3
	Legs	7.1
Postoperative phase		
Age at ICU admission in days (median, range)		31 (10–53)
Duration of ICU stay in days (median, range)		8 (2–36)
Total duration of hospital stay in days (median, range)		18 (7–46)
Postoperative need for mechanical ventilation (% yes)		64.3
Duration of mechanical ventilation in days (median, range)		6.5 (4–11)
**(b). Group 2–Major surgery** ***N** **=*** **10**		
**General characteristics**		
Preterm born (*n*)		3
Total score surgical stress* (median, range)		8.5 (6–15)
Age at ICU admission (days, median, range)		1.5 (0–29)
Age during surgery (days, median, range)		3.5 (1–30)
Surgical diagnosis (*n*)	Diaphragmatic hernia	3
	Malrotation	2
	Oesophageal atresia	1
	Malignancy (sacrococcygeal teratoma)	1
	Bladder exstrophy	1
	Perforation of the ductus choledochus	1
	Omphalocele	1
Mechanical ventilation postoperatively (% yes)		70
**(c). Group 3–ECMO** ***N** **=*** **36**		
General characteristics		
Age at ICU admission in days (median, range)		0 (0–16)
Oxygenation Index* prior to ECMO treatment (median, range)		42 (21–106)
Age at start ECMO treatment in h (median, range)		24 (5–398)
ECMO duration in h (median, range)		125 (53–369)
Duration of mechanical ventilation in days (median, range)		11 (2–70)
Surgery in the first months of life (*n*, %)		6 (17)
Diagnosis (%)	Meconium aspiration syndrome (*n*, %)	23 (64)
	Congenital diaphragmatic hernia (*n*, %)	6 (17)
	Sepsis (*n*, %)	2 (6)
	Persistent pulmonary hypertension of the newborn (*n, %*)	3 (8)
	Pneumonia (*n*, %)	1 (3)
	Other (*n*, %)	1 (3)
**(d). Group 4–Preterm born children** ***N** **=*** **19**		
**General characteristics**		
Ethnicity (Western European %)		68.4
Number of painful procedures per day* (median, range)		12 (4–18)
CRIB score (median, range)		4 (0–8)
Age at ICU admission in days (days, median, range)		0 (0–0)
Duration of ICU stay in days (days, median, range)		15 (4–63)
Duration of mechanical ventilation (days, median, range)		4 (2–26)
**(e). Group 5–NAS** ***N** **=*** **15**		
**Birth characteristics**		
Prematurely born (less than 37 weeks of gestation) (*n*, %)		4 (27%)
Apgar scores after 1 min (median, IQR)		9 (7–9)
Apgar scores after 5 min (median, IQR)		10 (9–10)
Apgar scores after 10 min (median, IQR)*		10 (10–10)
Born in our Hospital (*n*, %)		15 (100)
Intensive care admission (*n*, %)		3 (20)
Length of stay, in days (median, IQR)		17 (11–22)
**NAS**		
NAS (Finnegan score ≥8) (*n*, %)		14 (93)
**Demographic characteristics**		
West-European (*n*, %)		8 (53)
Caregiver	Adopted/foster parents (*n*, %)	13 (87)
	With relatives (grandmother) (*n*, %)	3 (23)
	Biological parents (*n*, %)	2 (13)
Education level of the child	Special primary education (*n*, %)	2 (13)
	Primary education (*n*, %)	4 (27)
	Lower vocational education (*n*, %)	5 (33)
	Intermediate vocational education (*n*, %)	3 (20)
	Higher vocational education (*n*, %)	1 (7)

(a). *Based on *n* = 9 due to missing data.

(b). *The surgical stress score measures the severity of surgical stress in neonates and has a range from 3–22, for more information see van Dijk et al. (17).

(c). *Oxygenation index is a calculation to measure the fraction of inspired oxygen (FiO2) and its usage within the body.

*Based on n = 34 due to missing data*.

*(d). CRIB: Clinical Risk Index for Babies, IV: intravenous*.

*Measured in the first 14 days, presented as mean per subject per day. Based on n = 14 due to missing data.

*(e)IQR, Interquartile range*.

*Apgarscore (after 10 min) was not scored for one child.

The numbers of children included per group as well as the age and gender distribution are presented in the Table 3. Moreover a summary of the results per outcome measure is shown in this Table 3.

**Table 3:** Overview of background characteristics and statistically significant results per group.

	**Group 1**		**Group 2**		**Group 3**		**Group 4**		**Group 5**	
	**GCMN**		**Surgery**		**ECMO**		**PRETERM**		**5 NAS**	
	**Cases**	**Controls**	**Cases**	**Controls**	**Cases**	**Controls**	**Cases**	**Controls**	**Cases**	**Controls**
***Number of cases***	*14*	*42*	*10*	*10*	*36*	*64*	*19*	*22*	*15*	*71*
**Mean age at inclusion (SD)**	12.3 (2.1)	11.6 (2.4)	15.5 (14.5–17.0)^*^	15.1 (14.0–17.0)^*^	11.1 (2.4)	11.1 (1.7)	10.2 (0.4)	10.4 (0.8)	14.2 (3.2)	11.7 (2.5)
**% boys**	64.3	52.4	80.0	60.0	47.2	43.0	68.2	68.4	26.7	42.3
**Neuropsych. functioning**	Not conducted^**^		**NS**		ECMO group worse on memory test		**NS**		NAS groups worse on several domains	
Results NEPSY					Lower total score on narrative memory; *p =* 0.001 *N =* 28 vs. *N =* 56				Worse performance on response set, word generation, arrows and geometric puzzles; *p =* 0.002 *N =* 12 vs. *N =* 68	
**Detection/pain thresholds**	**NS**		**NS**		ECMO group less sensitive for cold detection		**NS**		**NS**	
Results TSA-II					Mean cold detection (SD) EMCO 29.9 (1.4) vs. 30.6 (0.8) in controls using MLI; p < 0.01 *N =* 36 vs. *N =* 62					
**Structural MRI**	Thicker cortex left rostral middle frontal pole in GCMN		**NS**		**NS**		**NS**		**NS**	
Results T1 MRI	Thicker cortex left rostral middle frontal pole of 954.52 mm^2^, additionally corrected for age and gender *N =* 13 vs. *N =* 30									
**Functional MRI**	More parietal and occipital brain activation in GCMN		Less occipital brain activation in surgery group		**NS**		***Not conducted***		Less frontal brain activation in NAS group	
Results fMRI	Increased activation bilaterally in the parietal and occipital lobe *N =* 10 vs. *N =* 25		Less activation in the lateral occipital cortex *N =* 10 vs. *N =* 9						Less activation in one cluster consisting of the frontal pole *N =* 9 vs. *N =* 48	

* *Data presented in median (range)*.

** *Due to the relatively wide age range and small sample size in this group*.

*NS means no statistically significant differences between experimental cohort group and controls (after correction for multiple testing)*.

*GCMN, giant congenital melanocytic naevus; ECMO, extracorporeal membrane oxygenation; MLI, Method of Limits; NAS, neonatal abstinence syndrome*.

### Neuropsychological Functioning

ECMO-treated children performed statistically significantly worse on the memory task Narrative memory *p* = 0.001 (Table 4). Children of the NAS group performed statistically significantly worse on several domains including visiospatial, language, attention and executive functioning tests compared to controls (Geometric Puzzles *p* = 0.002; Response Set (more omission errors) *p* = 0.002, Word Generation *p* = 0.002, and Arrows *p* = 0.002; Table 4). Children of the Major Surgery group and the Preterm born children showed no differences compared to controls.

**Table 4:** Neuropsychological outcome.

**NEPSY-II Subtests**		**Group 3–ECMO**			**Group 5–NAS**			
		**ECMO *N =* 36**	**Controls *N =* 64**	*P*-value^*^	**NAS *N =* 12**	**Controls *N =* 68**	*P*-value^*^	*P*-value^**^
**Attention and executive functioning**								
Auditory Attention median (IQR)	Commission *errors*	0 (0–0)	0 (0–0)	0.71	0 (0–2)	0 (0–0)	0.17	0.43
	Omission *errors*	0 (0–1)	0 (0–1)	0.45	0 (0–4)	0 (0–1)	0.46	0.06
	Inhibitory *errors*	0 (0–0)	0 (0–0)	0.09	0 (0–0)	0 (0–0)	0.30	0.49
Response set median (IQR)	Commission *errors*	1 (1–3)	2 (0–4)	0.82	2 (0–5)	2 (0–4)	0.40	0.18
	Omission *errors*	3 (1–6)	3 (2–5)	0.79	4 (2–6)	3 (1–5)	0.18	**0.002**
	Inhibitory *errors*	0 (0–1)	0 (0–1)	0.92	0 (0–2)	0 (0–1)	0.74	0.24
**Language**								
Word Generation total score, median (IQR)		32 (25–40)	35 (27–40)	0.22	30 (25–35)	35 (27–41)	0.15	**0.002**
**Memory and learning**								
Memory for Faces total score, median (IQR)		12 (11–13)^§^	12 (10–13)	0.54	12 (10–13)	12 (10–13)	0.84	0.94
Memory for Faces Delayed total score, median (IQR)		12 (10–14)	12 (10–14)	0.99	13 (9–13)	12 (10–14)	0.75	0.29
Narrative Memory^§§^ total score, median (IQR)	Free recall	18 (14–24)	24 (20–26)	**0.001**				
	Free and cued recall	22 (19–25)	26 (22–29)	**0.001**	25 (20–29)	26 (22–29)	0.74	0.54
	Recognition	14 (14–15)	15 (15–16)	**0.001**	15 (14–15)	15 (15–16)	0.26	0.31
**Sensorimotor functioning**								
Visuomotor Precision^§§^ *total errors*, median (IQR)		7 (1–13)	10 (4–22)	**0.05** ^***^	15 (5–46)	10 (4–22)	0.52	0.41
**Visuospatial processing**								
Arrows total score, median (IQR)		28 (26–32)	28 (26–30)	0.53	26 (20–32)	28 (26–31)	0.12	**0.002**
Geometric Puzzles total score, median (IQR)		30 (27–33)	30 (27–34)	0.58	27 (25–31)	30 (28–34)	**0.02**	**0.002**
Route Finding^§§^ total score, median (IQR)		9 (8–10)	9 (8–10)	0.81	8 (7–8)	9 (8–10)	**0.02**	0.33

* *P-values were derived from Mann-Whitney U-test*.

** *P-values were derived from ANCOVA tests adjusted for gender and age (additional analyses because of wider age range)*.

*** *Not significant after correction for multiple testing*.

§ *n = 35 due to missing data in one subject*.

§§ *ECMO n = 28 vs. n = 56 since 8 subjects in both groups conducted six subtests of the NEPSY-II (since they were older than 12 years of age), NAS; n = 6 vs. n = 56 since 6 cases and 12 controls conducted six subtests of the NEPSY-II (since they were older than 12 years of age)*.

*The minimum and maximum scores of these subtests are: Auditory Attention commission errors: 0–180, omission errors: 0–30, inhibitory errors 0–35, Response set commission errors: 0–180, omission errors: 0–36, inhibitory errors: 0–37, Word generation: 0–no maximum, Memory for faces: 0–16, Memory for faces delayed: 0–16, Narrative memory free and cued recall: 0–34, recognition: 0–16, Visuomotor precision: 0–382, Arrows: 0–38, Geometric puzzles: 0–40, and Route finding: 0–10 points*.

### Detection and Pain Thresholds

No differences in pain thresholds were found between the groups compared to their control groups. With respect to detection thresholds the ECMO survivors (group 3) were less sensitive for the detection of cold compared to controls; mean (SD) cases 29.9 (1.4) vs. controls 30.6 (0.8); *P* < 0.01. Children of the GCMN (group 1), major surgery (group 2), preterm born (group 4), and NAS case groups (group 5) showed no statistically differences compared to controls.

### Structural Imaging Results

**Figure 2:**
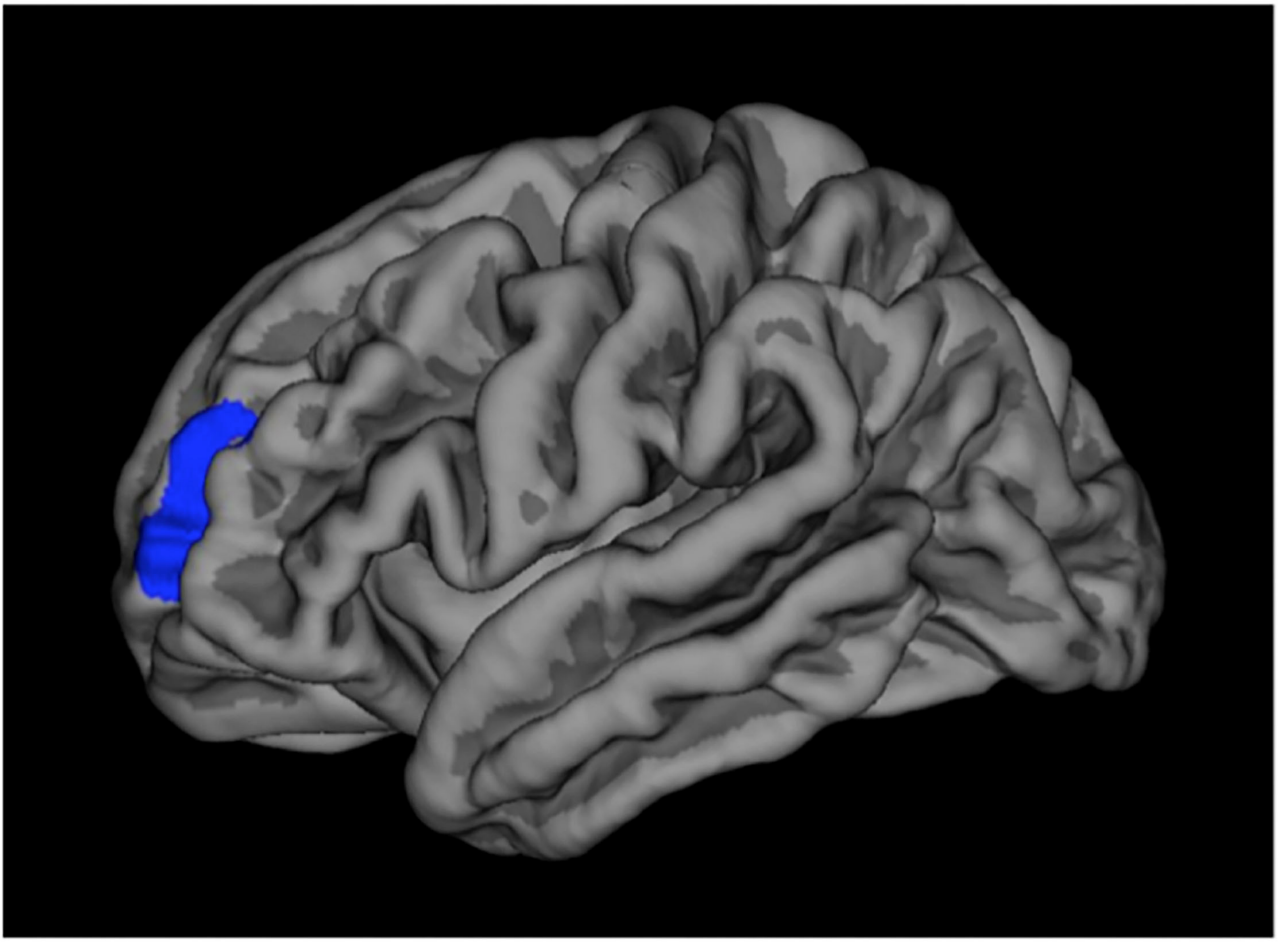
Cortical thickness.

In GCMN children (group 1) we found a significantly thicker cortex compared to controls in the left rostral-middle-frontal pole, corrected for age and gender and multiple testing and involved a region with a surface extent of 954.52 mm^2^ (Figure 2). We found no other statistically significant differences in brain morphology in this or the other groups compared to their controls.

Differences in cortical thickness in the left hemisphere in which GCMN children (group 1) have a statistically significant thicker cortex compared to controls in the rostral-middle-frontal pole (region marked in blue).

### Correlations With Morphine Exposure

With respect to morphine exposure in the ECMO cohort and the NEPSY results (*n* = 22/26 depending on the subtest), only the subtest Narrative Memory Recognition was significantly correlated (Spearman's coefficient 0.42, *p* = 0.05). No statistically significant correlations between total morphine exposure (*n* = 26) and detection thresholds (MLI and MLE), pain thresholds, or NRS scores were found. Moreover, no statistically significant correlations between total morphine exposure (*n* = 16) and brain volumes were found, and the positive and negative correlation coefficients indicated weak to moderate associations varying between <0.01 and 0.49.

### Functional Imaging Results

**Figure 3:**
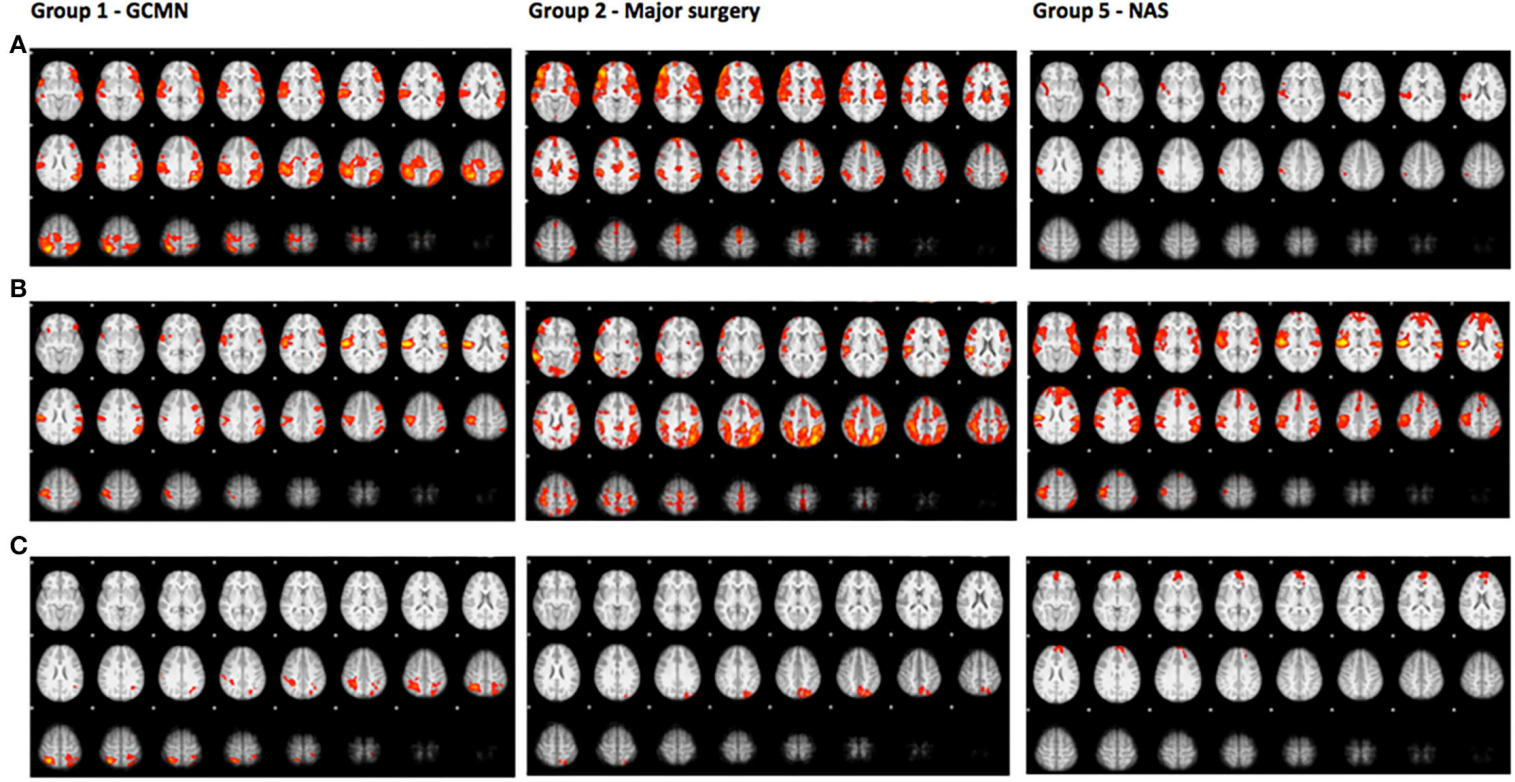
Brain activation during pain.

A direct comparison of brain activation during pain in group 1 (GCMN) revealed statistically significant increased activation bilaterally in the parietal and occipital lobe in the GCMN children. After correction for gender and age the intensity of the activation was reduced in both groups and no longer significantly different. A direct comparison of statistically significant brain activation during pain in group 2 (major surgery) revealed significantly more brain activation in mainly the lateral occipital cortex in the control group compared to the case group. Due to the small sample size additional correction for age and gender was not conducted. In group 5 (NAS), a direct comparison revealed statistically significantly greater brain activation in one cluster consisting mainly of the frontal pole in the control group compared to the cases, but the significance disappeared after correction for age and gender (Figure 3 and Table 5). We found no statistically significant differences in brain activation during pain between the ECMO group and controls. Because of poor image quality due to movement, no fMRI analyses could be conducted in the Preterm born group.

**Table 5:** Areas of activation during pain.

**Cluster size (voxels)**	***P*-value**	**MNI coordinates local maxima (mm)**			***Z*-value**	**Anatomical area**
		***X***	***Y***	***Z***		
**Group 1–GCMN**						
**Mean activation cases**						
16,872	<0.0001	30	−54	56	5.22	Superior parietal lobule (R)
		34	−52	62	4.94	
		38	−42	44	4.69	
		36	−64	58	4.90	Lateral occipital cortex (R)
		42	−58	56	4.61	
		58	10	−14	4.69	Temporal pole (R)
10,579	<0.0001	−40	−58	30	4.69	Angular gyrus (L)
		−48	−56	56	4.47	
		−34	−60	42	4.66	Lateral occipital cortex (L)
		−62	−16	30	4.54	Postcentral gyrus (L)
		−70	−30	4	4.46	Superior temporal gyrus (L)
		−66	−30	4	4.43	
5,355	<0.0001	−36	22	−30	3.95	Temporal pole (L)
		−52	16	−12	3.74	
		−44	6	40	3.85	Middle frontal gyrus (L)
		−52	32	0	3.79	Inferior frontal gyrus (L)
		−54	22	10	3.75	
		−46	18	10	3.70	
**Mean activation controls**						
6,129	<0.0001	52	−16	16	5.48	Central opercular cortex (R)
		64	−16	16	5.08	
		52	−6	10	4.58	
		44	−30	52	4.36	Postcentral gyrus (R)
		56	22	−16	4.25	Temporal pole (R)
		36	6	10	3.79	Insular cortex (R)
3,580	0.002	−58	−22	18	4.62	Central opercular cortex (L)
		−52	−48	28	4.57	Submarinal gyrus (L)
		−52	−48	36	4.23	
		−46	−58	32	4.40	Angular gyrus (L)
		−64	−54	28	3.57	
		−62	−60	38	3.44	Lateral occipital cortex (L)
2,942	0.005	−50	26	−24	3.86	Temporal pole (L)
		−46	22	−28	3.70	
		−38	10	36	3.85	Middle frontal gyrus (L)
		−50	12	50	3.83	
		−54	14	46	3.81	
		−52	18	14	3.83	Inferior frontal gyrus (L)
**Direct comparison (mean cases** **>** **mean controls)**						
2,807	0.006	36	−64	58	4.84	Lateral occipital cortex (R)
		26	−60	68	3.87	
		20	−64	68	3.71	
		30	−54	56	4.57	Superior parietal lobule (R)
		32	−54	62	4.35	
		26	−48	44	4.04	
2,073	0.026	−30	−72	60	3.82	Lateral occipital cortex (L)
		−28	−68	58	3.72	
		−26	−72	50	3.66	
		−36	−72	56	3.54	
		−38	−48	64	3.42	Superior parietal lobule (L)
		−36	−46	68	3.40	
**Group 2–Major surgery**						
**Mean activation cases**						
21,434	<0.0001	−10	−8	24	3.92	Midline, cingulate gyrus
		−46	−52	38	3.89	Angular gyrus (L)
		−46	−50	32	3.76	
		−38	−82	−44	3.74	Cerebellum (L)
		−50	−22	−14	3.70	Middle temporal gyrus (L)
		−52	12	−12	3.63	Temporal POLE (L)
20,233	<0.0001	52	42	0	4.50	Frontal pole (R)
		40	38	−4	4.44	
		52	46	−4	4.41	
		50	40	−10	4.22	
		48	52	8	4.12	
		66	8	−2	4.13	Superior temporal gyrus (R)
**Mean activation controls**						
42,699	<0.0001	−36	−70	42	4.53	Lateral occipital cortex (L)
		−32	−76	38	4.25	
		66	−44	−6	4.28	Middle temporal gyrus (R)
		54	−46	−6	4.21	
		58	−48	−4	4.17	
		−48	−50	36	4.16	Supramarginal gyrus (L)
**Direct comparison (mean controls** **>** **mean cases)**						
1,747	0.030	−28	−80	42	3.67	Lateral occipital cortex (L)
		−22	−70	58	3.06	
		−46	−82	32	3.05	
		−36	−82	46	2.98	
		−26	−86	30	2.92	
		−2	−72	44	3.40	Precuneus cortex (L)
**Group 5–NAS**						
**Mean activation cases**						
2,767	0.013	66	−32	28	3.63	Supramarginal gyrus (R)
		60	−38	26	3.48	
		38	−6	−12	3.42	Insula (R)
		38	−14	−6	3.16	
		40	−26	18	3.40	Parietal operculum cortex (R)
		38	−16	−10	3.18	Planum polare (R)
**Mean activation controls**						
14,473	<0.0001	−60	−24	18	5.12	Parietal operculum cortex (L)
		−52	−48	30	4.57	Supramarginal gyrus (L)
		−52	30	−18	4.52	Frontal pole (L)
		−56	−24	−14	4.49	Middle temporal gyrus (L)
		−50	26	−22	4.38	Temporal pole (L)
		−60	−58	40	4.36	Lateral occipital cortex (L)
12,820	<0.0001	46	−18	14	6.00	Central opercular cortex (R)
		66	−16	14	4.94	
		36	6	10	4.25	
		50	24	−20	4.76	Temporal pole (R)
		54	22	−18	4.74	
		70	−34	−4	4.42	Middle temporal gyrus (R)
7,226	<0.0001	−2	70	26	4.79	Frontal pole (L)
		−20	66	22	4.67	
		−2	66	30	4.62	
		−2	62	38	4.08	
		20	74	16	4.20	Frontal pole (R) Frontal pole (R)
		2	74	14	4.06	
**Direct comparison (mean controls** **>** **mean cases)**						
2,604	0.017	4	60	−4	3.80	Frontal pole (R)
		6	66	2	3.42	
		2	68	30	3.24	
		−6	64	28	3.52	Frontal pole (L)
		−8	68	22	3.37	
		−8	54	6	3.22	Paracingulate gyrus (L)

*Areas of activation during pain (46°C vs. baseline) with cluster size, Z-values of the local maximum, Montreal Neurological Institute (MNI) coordinates, and the anatomical area of the local maximum (Harvard-Oxford Cortical Structural Atlas)*.

*R, Right; L, Left*.

The axial slices show colored areas of activation during pain in the cases (a), the control group (b) and the direct comparison between both groups (c; cases > controls in group 1 and controls > cases in group 2 and group 5) using a cluster significance threshold of *p* < 0.05.

## Discussion

While previous studies focused on one specific cohort such as very preterm born children (24) or conducted a follow-up study among children exposed to very high supratherapeutic amounts of opioids (30 mcg/kg/h) (25), our study covers the continuum from no pain to intense pain and from no opioid exposure to very high opioid exposure in the presence or absence of anesthetics in five unique groups with a wide age range from children to young adults. We found no major long-term effects (between 8 to 19 years after exposure) on pain sensitivity, brain functioning during pain and brain morphology. Nevertheless, the memory performance of ECMO survivors and the neuropsychological performance of children exposed to opioids *in utero* were worse compared to controls.

Previous studies in animals suggest neurotoxic effects of early exposure to pain, opioids and anesthetics separately from each other (3–9, 26–34), while opioids were found neuroprotective if administrated in the presence of pain (3, 11, 12). Interestingly, studies in humans show contradictory results (1). Possibly since children are exposed to a combination of pain, opioids and anesthetics. In order to unravel the potential negative long-term effects of those three elements we studied the continuum from no pain to intense pain and from no opioid exposure to very high opioid exposure in the presence or absence of anesthetics in five unique study groups.

In the group of children with GCMN (group 1), extensive tissue damage (median 18% BSA) and associated intense pain in combination with very high exposure to opioids and exposure to anesthetics was associated with more parietal and occipital brain activation during pain compared to healthy controls. Less extensive tissue damage in the group children that required major non-cardiac thoracic or abdominal surgery and received usual amounts of opioids (10.0–11.2 mcg/kg/h in the first 24 postoperative h) combined with exposure to anesthetics (group 2) showed less occipital brain activation during pain compared to healthy controls. It is interesting that the differences in brain activation during pain between group 1 and 2 and their controls were not specifically located in the pain centers of the brain, but rather in sensory regions. Since primary cortical areas typically develop earlier than secondary or tertiary brain regions (35), it is possible that early exposure to pain, opioids and anesthetics resulted in activity-dependent neuronal changes in the primary and secondary sensorimotor cortical regions. We were surprised to find more brain activation in group 1 (GCMN) and less in group 2 (major surgery) in the same occipital brain region. A possible explanation could be that mean postnatal age differed between these groups during our follow-up program, but also during the moment of surgery early in life (Table 2). Another potential explanation could be that children in group 1 experienced more breakthrough pain due to the extensive tissue damage as reflected by the high need for opioids. This difference in neonatal pain and opioid exposure could have caused the difference between groups since it is known that the effects of opioids are different when given in the absence or presence of pain, at least in rodents (3, 11, 12). Unfortunately, we only have detailed information regarding opioid exposure in the first 24 h in group 2. However, we expect the postoperative course in group 1 as more painful than group in 2 which associated higher opioid exposure in group 1. With regard to the effects of anesthetic exposure, our results are in line with the findings of the GAS study indicating that general anesthesia with sevoflurane does not alter neurodevelopmental outcome in children (36, 37).

Prolonged continuous opioid exposure in the absence of major pain, as seen in ECMO-treated newborns (group 3), induced no alterations in brain morphology. We did find hyposensitivity for cold detection, although prolonged use of opioids even in the most critically ill newborns did not result in an altered response of the central nervous system–at least as evaluated by fMRI. No statistically significant correlations between total morphine exposure and detection and pain thresholds, NRS scores, or brain volumes were found. ECMO survivors performed statistically significantly worse on specific memory subtests compared to healthy controls. When a subtest result indicates statistically significant worse functioning, the worse functioning is likely associated with clinically significant difficulties in daily life and does warrant further investigation. The findings for the ECMO group were indeed in line with our own experience at the ECMO survivors' outpatient follow-up clinic (38). The worse functioning is important from a neurodevelopmental point of view and probably unrelated to pain and opioids, although one memory subtest was significantly correlated to morphine exposure. A common neurodevelopmental pathway seems to exist across various types of neonatal critical illness, in which early hippocampal alterations result in long-term memory deficits (39). Moreover, vasoactive medication during neonatal life seems to be associated with verbal and visiospatial memory later in life, suggesting an effect of early cerebral hypoperfusion (40).

Our cohort of preterm born children exposed to low dosages of opioids in the absence of tissue damage and substantial pain (group 4) has been comprehensively studied in two other follow-up studies in our department (41, 42). In line with these two previous studies, in the present study we did not find major negative effects of prematurity, procedural pain and routine preemptive morphine administration on neuropsychological functioning. Moreover, we did not find an influence of morphine consumption on pain sensitivity, in contrast to a study by Buskila et al. in 60 preterm born children compared to 60 controls at age 12–18 years, which, however, did not report the amount of neonatal morphine exposure (43). The contrast between both studies might perhaps be explained by a higher morphine exposure in the study of Buskila et al. We found no statistically significant differences in brain volumes between preterm born children and healthy controls, indicating no major clinically relevant influence of pain and opioid exposure on brain morphology. This is in contradiction to previous studies in preterm born morphine-exposed children that found differences in head circumference, cortical thickness, brain microstructure, and brain functioning at term-equivalent age, and in childhood (13, 16, 44–46). A possible explanation for differences between studies is that any reductions in brain volume at term-equivalent age had disappeared over time due to the inherent plasticity of the brain. Additionally, the children in our cohort had received low doses of opioids, while other cohorts were exposed to higher dosages (25).

Since animal studies describe different outcomes of opioid exposure when given in the absence or presence of pain, we added a unique group of individuals exposed to synthetic opioids *in utero* (group 5). We did not find differences between this group and healthy controls with respect to pain sensitivity or brain morphology. However, we found worse neuropsychological functioning, in line with cognitive, memory and behavioral problems in rodents after exposure to opioids in the absence of pain (7–9). This is also in line with negative behavioral and cognitive outcome in humans after opioid exposure *in utero* (47). We also found less frontal brain activity in this group during pain. The frontal brain region is associated with attention and executive functioning. Taken together, high exposure to opioids in the absence of pain appears to have the most negative effects, especially on neuropsychological functioning (ECMO group and *in utero* exposed group). However, in these particular circumstances several factors in both groups may also have contributed to worse neuropsychological outcomes, such as the illness severity in group 3 and genetic and psychosocial factors and polydrug abuse of mothers of the children in group 5. Moreover, most of the children in group 5 (93%) were exposed to phenobarbital after birth to treat the neonatal abstinence syndrome. This exposure could have influenced our results since phenobarbital is a drug with potential neurotoxic properties and has been associated with long-term behavioral problems in rats (48).

Animal studies hamper from a methodological disadvantage since the painful stimuli are not similar to those in humans, therefore animal studies using stimuli mimicking the human situation are needed (49). Moreover, experimental animals often receive supratherapeutic high dosages of drugs and mostly for prolonged periods of time and in the absence of pain (50, 51). Furthermore, the manifestation of peak synaptogenesis may occur at different periods among species, and the window of vulnerability between animals and humans may be different (52).

Our study does provide a proof-of-principle to assess the feasibility of evaluating possible long-term neurodevelopmental effects of early exposure of pain, opioids, and anesthesia. The neurodevelopmental effects can be evaluated through the examination of neuropsychological functioning, thermal detection and pain thresholds and high-resolution structural and task-based functional magnetic resonance imaging during pain. Our comprehensive follow-up study should not be considered as definitive proof due to specific limitations. Notably, while we conducted several important and feasible subtests, we did not focus on other potential long-term effects such as differences in quality of life or behavior. Our study can serve as a springboard for future studies evaluating this important topic including other relevant outcomes such as quality of life and behavior as well. Moreover, although we evaluated five unique groups of children recruited from well-documented neonatal cohorts in a systematic way, our sample size per subgroup was relatively low. Some subgroups were underpowered and too small to draw firm conclusions on outcome. Besides, the sample size did not allow to correct for possible confounders other than age and gender. Confounders such as social economic status, nutrition, level of parental education, comorbidity or exposure to other drugs than opioids or anesthetic agents could have played a role in our findings. Since controls were recruited by asking all participating families whether they could recommend a child who would be willing to participate, selection bias is a possibility. However, we tried to overcome this by additional random recruitment from schools. Moreover, possible selection bias has occurred because children with the most severe neurological and cognitive outcomes did not participate. While most data were prospectively collected during neonatal life, some variables such as illness severity scores and length of anesthesia were not routinely collected at that time. However, the included children had all been exposed to early severe pain, opioid-related substances or anesthetics.

In conclusion, we show no major effects that remain in the human brain after neonatal pain, opioid or anesthetic exposure some 8–19 years later. We conclude that besides specific neuropsychological effects in humans that warrant further investigation, we did not detect major clinical relevant effects with respect to thermal and pain sensitivity, brain functioning during pain or brain morphology. However, future studies with larger sample sizes are needed to confirm our findings or to detect less pronounced effects of neonatal pain, opioid or anesthetic exposure. We believe that pain treatment is extremely important and that the use of low dosages opioids for procedural pain or intense pain because of major tissue damage seem not harm the brain in humans dramatically later in life.

## Data Availability Statement

The raw data supporting the conclusions of this article will be made available by the authors, without undue reservation.

## Ethics Statement

The studies involving human participants were reviewed and approved by the Institutional Review Board at the Erasmus MC (MEC-2010-299). Written informed consent to participate in this study was provided by the participants' legal guardian/next of kin.

## Author Contributions

GB: literature search, recruitment, data collection, data analysis, data interpretation, writing up the first draft version of the manuscript, and approval of the final manuscript as submitted. DT: study design, data interpretation, critical revision of the manuscript for important intellectual content, and approval of the final manuscript as submitted. JG and AL: data interpretation, critical revision of the manuscript for important intellectual content, and approval of the final manuscript as submitted. HE: assistance in the structural imaging analyses, data interpretation, critical revision of the manuscript for important intellectual content, and approval of the final manuscript as submitted. TW: study design, supervision of MRI data collection and analyses, data interpretation, critical revision of the manuscript for important intellectual content, and approval of the final manuscript as submitted. MD: study design, supervision of TSA and NEPSY analyses, data interpretation, critical revision of the manuscript for important intellectual content, and approval of the final manuscript as submitted. All authors contributed to the article and approved the submitted version.

## Funding

This study was supported by the Dutch Research Council: ZonMw Priority Medicines for Children Grant 40-41500-98.9020.

## Conflict of Interest

The authors declare that the research was conducted in the absence of any commercial or financial relationships that could be construed as a potential conflict of interest.

## Publisher's Note

All claims expressed in this article are solely those of the authors and do not necessarily represent those of their affiliated organizations, or those of the publisher, the editors and the reviewers. Any product that may be evaluated in this article, or claim that may be made by its manufacturer, is not guaranteed or endorsed by the publisher.

## Abbreviations

BSA: Body surface area
ECMO: Extracorporeal membrane oxygenation
FMRI: Functional Magnetic Resonance Imaging
GCMN: Giant congenital melanocytic naevus
MRI: Magnetic Resonance Imaging
NAS: Neonatal abstinence syndrome
TSA-II: Thermal Sensory Analyzer-II.
