# Supplementary material for: Neonatal Pain, Opioid, and Anesthetic Exposure; What Remains in the Human Brain After the Wheels of Time?
Source: Front Pediatr. 2022 May 11;10:825725. doi: 10.3389/fped.2022.825725 (PMC9132108; doi:10.3389/fped.2022.825725)
Supplement: Supplementary file 1 [file Presentation_1.pptx]

## Slide 1
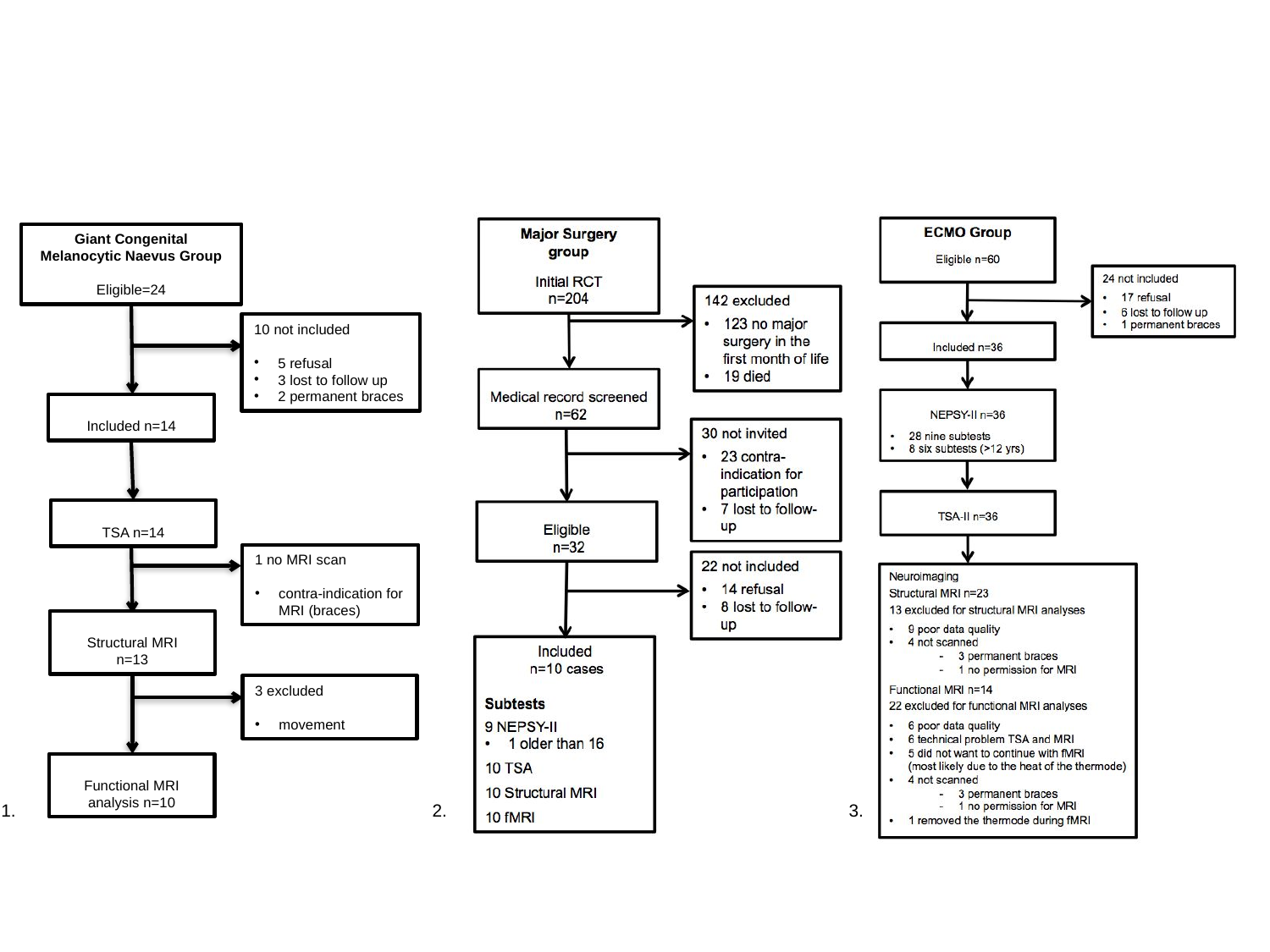

Giant Congenital Melanocytic Naevus Group
Eligible=24
10 not included
5 refusal
3 lost to follow up
2 permanent braces
TSA n=14
1 no MRI scan
contra-indication for MRI (braces)
Structural MRI
n=13
3 excluded
movement
Functional MRI analysis n=10
1.
Included n=14
2.
3.
