# Supplementary material for: Neonatal Pain, Opioid, and Anesthetic Exposure; What Remains in the Human Brain After the Wheels of Time?
Source: Front Pediatr. 2022 May 11;10:825725. doi: 10.3389/fped.2022.825725 (PMC9132108; doi:10.3389/fped.2022.825725)
Supplement: Supplementary file 2 [file Presentation_2.pptx]

## Slide 1
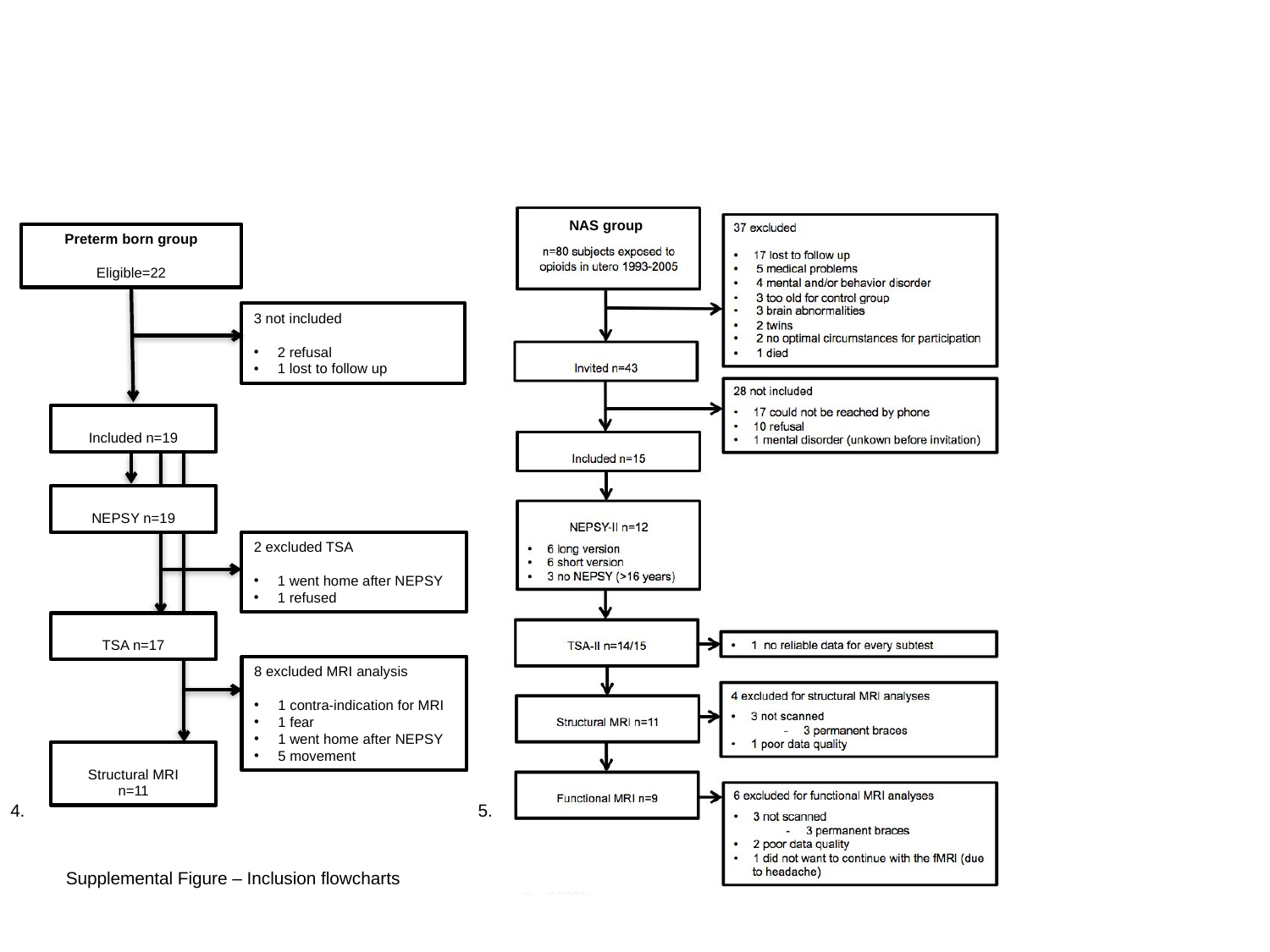

NAS group
Preterm born group
Eligible=22
3 not included
2 refusal
1 lost to follow up
Included n=19
8 excluded MRI analysis
1 contra-indication for MRI
1 fear
1 went home after NEPSY
5 movement
Structural MRI
n=11
4.
NEPSY n=19
2 excluded TSA
1 went home after NEPSY
1 refused
TSA n=17
5.
Supplemental Figure – Inclusion flowcharts
