# Supplementary figures and images for: Neonatal Pain, Opioid, and Anesthetic Exposure; What Remains in the Human Brain After the Wheels of Time?
Source: Front Pediatr. 2022 May 11;10:825725. doi: 10.3389/fped.2022.825725 (PMC9132108; doi:10.3389/fped.2022.825725)

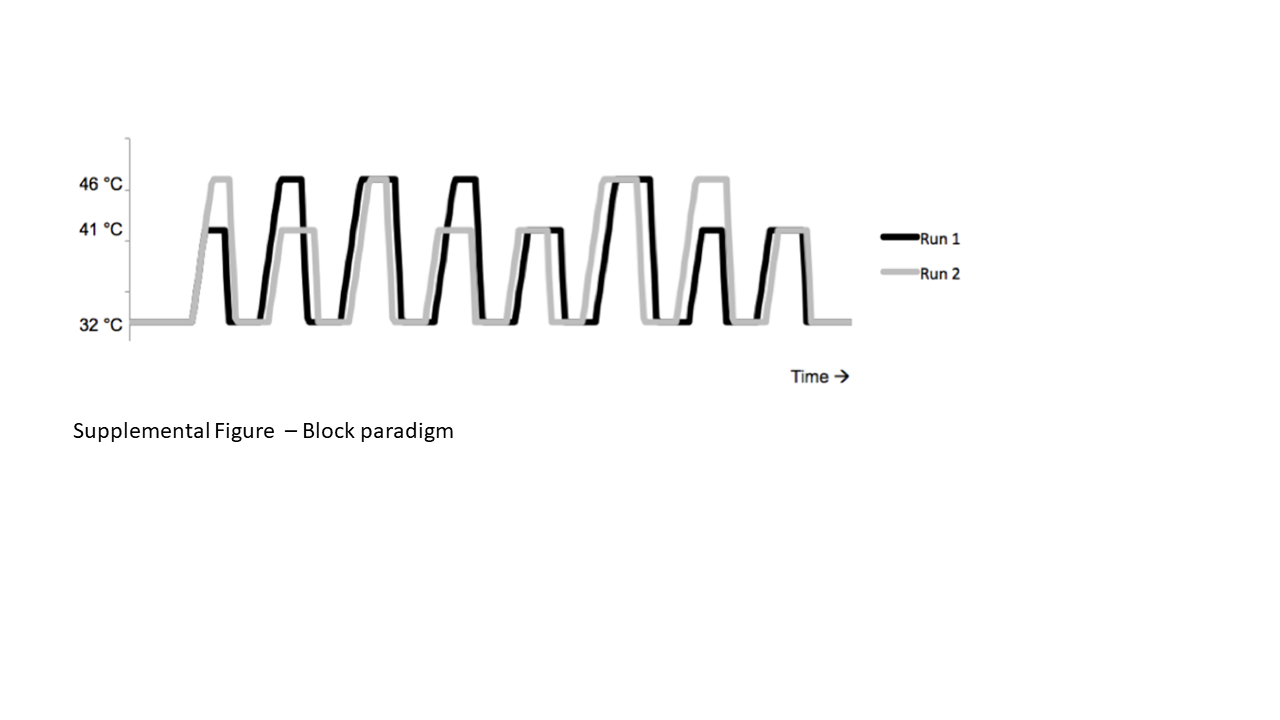

Supplement: Supplementary file 3 [file Image_2.tif]
