# Supplementary material for: Neonatal Pain, Opioid, and Anesthetic Exposure; What Remains in the Human Brain After the Wheels of Time?
Source: Front Pediatr. 2022 May 11;10:825725. doi: 10.3389/fped.2022.825725 (PMC9132108; doi:10.3389/fped.2022.825725)
Supplement: Supplementary file 4 [file Data_Sheet_1.docx]

**SUPPLEMENTAL FILE - MATERIAL AND METHODS**

**Examination of detection and pain thresholds**

To determine detection- and pain thresholds we used the Thermal Sensory Analyzer-II (TSA-II, Medoc Advanced Medical systems, Israel). The TSA-II is a precise, computer-controlled device capable of generating and recording a response to a highly repeatable thermal stimulus over a range of 0 °C to 50 °C. A Peltier-based contact thermode (30 x 30 mm) was placed at the thenar eminence of the non-dominant hand to apply cold or heat to the child’s skin. We determined detection- and pain thresholds using a standardized protocol. After explaining the test we first determined the children’s detection- and pain thresholds for cold and warmth using the reaction time dependent Method of Limits (MLI). The test started at a baseline temperature of 32 °C, which was then steadily linearly decreased at a rate of 1 °C/sec. The child was asked to press the button as soon as the cold stimulus was felt. After pressing the button, the stimulus reversed to the baseline temperature of 32 °C with a rate of 1 °C/sec. We repeated this five times. The first two stimuli served as rehearsal stimuli. The detection threshold was calculated as the mean value of the last four stimuli. Next, the temperature was steadily increased at a linear rate of 1 °C/sec to determine the detection threshold for warmth using the same method. Second, the MLI was applied to determine the pain thresholds for cold and warmth. Starting again from a baseline temperature of 32 °C, the temperature was steadily decreased at a linear rate of 1.5 °C/sec. The child was asked to press the button when the cold sensation started to feel painful. Now also, the temperature reversed to the baseline temperature with a rate of 10.0 °C/sec. This was repeated four times. The last four temperatures obtained were used to calculate the mean pain threshold. Next, the pain threshold for warmth was determined in the same manner. When a child did not press the button before 0 °C or 50 °C, the test automatically terminated. Furthermore we determined the detection thresholds for cold and warmth again, but now using the reaction time independent Method of Levels (MLE). The researcher told the children that the thermode could either become cold, or would not change in temperature. The first thermal stimulus was 3.0 °C below the baseline temperature of 32.0 °C. Following each thermal stimulus the researcher asked the child if the thermode become cold or not. Dependent on the child’s response, the next stimulus was increased or decreased in temperature. The test terminated when the step size of the stimulus had decreased to a level of 0.1 °C. The warm detection threshold was determined in the same manner starting with a stimulus temperature of 3.0 °C above the baseline temperature.

**Image acquisition**

MR images were acquired on a 3 Tesla scanner (General Electric Discovery MR750, Milwaukee, MI, USA) using an 8-channel head coil. We obtained high-resolution structural T_1_-weighted images using an inversion recovery fast spoiled gradient recalled (IR-FSPGR) sequence with the following parameters: TR=10.3 ms, TE=4.2 ms, TI=350 ms, NEX=1, flip angle=16°, readout bandwidth=20.8 kHz, matrix 256x256, imaging acceleration factor of 2, phase encoding was anterior to posterior, and an isotropic resolution of 0.9x0.9x0.9 mm^3^. The scan time for the structural T_1_ MRI scan was 5 minutes and 40 seconds. We conducted two runs of a functional MRI paradigm using single-shot echo-planar imaging (EPI) T_2_*-weighted sequences in transverse orientation sensitive to blood oxygen level dependent (BOLD) contrast (parameters: TR/TE 2000/30 ms, flip angle 85°, 64x64 matrix with a field-of-view of 260x260 mm^2^; 39 slices and voxel sizes of 3.6x3.6x4.0 mm^3^). Scan time was 182 TRs (6 minutes 4 seconds) per run.

**Functional MRI Block paradigm**

The functional MRI (fMRI) component consisted of two runs and utilized a block paradigm. During each of these two runs the TSA-II thermode was applied to the thenar eminence of the non-dominant hand. During scanning the TSA-II thermode induced warm (41 °C) and painful stimuli (46 °C). These temperatures were derived from a previous study from our research group(1). Within each run, the temperature increased four times at a rate of 1.5 °C/sec from the baseline temperature of 32 °C to a warm temperature of 41°C and four times to a potentially painfully hot temperature of 46 °C. After each stimulus, the temperature decreased with 4.5 °C/sec back to baseline and stayed at the baseline temperature for 15 seconds before the increasing to the next warm or pain stimulus. The order and duration (8-16 seconds) of the stimuli was randomly determined at the beginning of the study and were different in both runs. In order to prevent anticipation to the stimuli, the order of warm and heat stimuli differed between the two runs. Figure 2 shows the block paradigm of the thermal stimuli for run 1 and run 2.

**Structural imaging analysis**

We used the FreeSurfer image analysis suite version 5.1.0 (<http://surfer.nmr.mgh.harvard.edu/>) for cortical reconstruction and volumetric segmentation. Freesurfer computes structural morphometric measures in an automated approach. These include bias field correction, skull stripping, cortical and subcortical segmentation, and cortical surface reconstruction (2). Each image was visually inspected and subjects with poor quality data were excluded. In subjects with small errors in the gray/white segmentation, control points, and white matter edits were added to identify and correct misclassified white matter regions. When the segmentation improved, the corrected images were used. Total brain volume and the volume of a priori selected pain related brain regions, including the thalamus, anterior cingulate cortex and insula (3), were compared between cases and controls using ANCOVAs correcting for age, gender, and total brain volume. Statistical analyses were performed using SPSS version 20.0. Evaluation of surface-based cortical thickness FreeSurfer was performed using the built-in program QDEC (2) with a smoothing filter of 10 millimeter. For the group analysis a general linear model (GLM) was fitted at each surface vertex. We corrected for age and gender and used a Monte Carlo correction (p<0.05) for multiple testing.

**Functional imaging analysis**

For functional MRI analyses (fMRI), we used a combination of Analysis of Functional Neuroimages (AFNI, http://afni.nimh.nih.gov/) (4) and FSL’s FMRIB’s Software Library (FSL 5.0, FMRIB Software Library; FMRIB, Functional Magnetic Resonance Imaging of the Brain; http://www.fmrib.ox.ac.uk/fsl/) (5). AFNI was used for slice timing and motion correction. Runs with more than 6 mm of motion (maximum displacement) were excluded from the analyses. Functional images for each individual were co-registered to their high-resolution T_1_ image and both functional and structural images were registered to the Montreal Neurological Institute (MNI) 152 atlas using FSL’s non-linear registration tool FNIRT. Finally, data were spatially smoothed using AFNI with an 8-mm full width at half-maximum Gaussian kernel (6). Following the preprocessing steps, single-subject analyses were performed using FMRIB's fMRI Expert Analysis Tool FEAT (http://www.fmrib.ox.ac.uk/fsl/feat5/index.html), comparable to a previous report of our study group (7). The time series for the pain runs were modeled using a block design. Design matrices were created for both runs using the data from each subject’s stimulus log file from the TSA. These matrices were created independently for each individual using an automated MATLAB program (MATLAB 7.1, The MathWorks Inc., Natick, MA, 2000). This modeled time series was convolved with the hemodynamic response function. Next, a general linear model was implemented using FMRIB's Improved Linear Model. The two within-subject runs were combined using a fixed effects model. The higher-level group analysis, which compared patients and controls for the contrast; 46 °C versus baseline, was performed using FMRIB's Local Analysis of Mixed Effects. We corrected for multiple comparisons using random Gaussian fields and significance was set at p<0.05 (two-tailed).

**References**

1. van den Bosch GE, van Hemmen J, White T, Tibboel D, Peters JW, van der Geest JN. Standard and individually determined thermal pain stimuli induce similar brain activations. Eur J Pain. 2013.

2. Fischl B, Salat DH, Busa E, Albert M, Dieterich M, Haselgrove C, et al. Whole brain segmentation: automated labeling of neuroanatomical structures in the human brain. Neuron. 2002;33(3):341-55.

3. Apkarian AV, Bushnell MC, Treede RD, Zubieta JK. Human brain mechanisms of pain perception and regulation in health and disease. Eur J Pain. 2005;9(4):463-84.

4. Cox RW. AFNI: software for analysis and visualization of functional magnetic resonance neuroimages. Comput Biomed Res. 1996;29(3):162-73.

5. Smith SM, Jenkinson M, Woolrich MW, Beckmann CF, Behrens TE, Johansen-Berg H, et al. Advances in functional and structural MR image analysis and implementation as FSL. Neuroimage. 2004;23 Suppl 1:S208-19.

6. White T, O'Leary D, Magnotta V, Arndt S, Flaum M, Andreasen NC. Anatomic and functional variability: the effects of filter size in group fMRI data analysis. Neuroimage. 2001;13(4):577-88.

7. White T, Hongwanishkul D, Schmidt M. Increased anterior cingulate and temporal lobe activity during visuospatial working memory in children and adolescents with schizophrenia. Schizophr Res. 2011;125(2-3):118-28.
